# Augmented reality in pelvic surgery: using an AR-headset as intraoperative radiation-free navigation tool

**DOI:** 10.1007/s11548-025-03462-6

**Published:** 2025-06-26

**Authors:** Vincent K. Schenk, Markus A. Küper, Maximilian M. Menger, Steven C. Herath, Tina Histing, Christof K. Audretsch

**Affiliations:** 1 Faculty of Medicine, Eberhard Karls University of Tübingen, Tübingen, Germany; 2 Department for Traumatology and Reconstructive Surgery, BG Trauma Center, University of Tübingen, Schnarrenbergstraße 95, 72076 Tübingen, Germany

**Keywords:** Augmented reality, Pelvic surgery, Trauma surgery, Surgical navigation, Guided surgery

## Abstract

**Purpose:**

The incidence of acetabular and pelvic fractures is rising significantly. Pelvic ring fractures rank as the sixth most common fractures in adults, with the majority occurring in the elderly. Due to complications related to surgical approaches, with rates of up to 31%, there is an increasing demand for minimally invasive surgical techniques. Augmented Reality (AR) has the potential to facilitate spatial orientation by a sophisticated user interface. The aim of this study was to develop an AR-based, radiation-free navigation system for pelvic fractures.

**Methods:**

The Microsoft® HoloLens 2 was used as the AR headset. The Unity® game engine was used for programming. Pelvic models from Sawbones® served as the model. Segmentation was performed using Slicer3D by Slicer Corporation. The symphysis and both anterior superior iliac spines were defined as anatomical reference points. Ten pelvic models were used for testing. A preoperatively defined drill trajectory was displayed to the surgeon. A total of 20 S1 screws and 19 S2 screws were placed using only AR navigation without visual access to the pelvic model. Screw placement was controlled using CT.

**Results:**

The matching process took an average of 3 min and 28 s. 18 out of 20 (90%) S1 screws and 3 out of 20 (15%) S2 screws were placed correctly. In most cases, no perforation occurred. The mean procedure time was 7 min for S1 screws and 5 min for S2 screws.

**Conclusion:**

Proper drilling was achieved by displaying the trajectories via AR, particularly for S1 screws, where a slightly wider drilling corridor was aimed for compared to S2 screws. No registration scan was necessary with our matching method. No intraoperative radiation was required.

## Introduction

The incidence of acetabular and pelvic fractures is rapidly increasing, making them the sixth most common fractures in adults [1]. This trend is caused by a rising number of fractures among individuals with an age of 60 years and older, while the incidence in younger patients has only slightly changed [2, 3]. In fact, in 2019, the incidence of acetabular and pelvic fractures in geriatric patients was tenfold higher when compared to a younger patient collective (43.9 per 100,000 vs. 4.3 per 100,000) [1]. Interestingly, younger patients typically suffer from high-impact trauma, such as traffic accidents or falls from a significant height, while the elderly are more prone to fractures caused by low-impact falls [3]. The latter are the result of age-related alterations of the bone metabolism such as osteoporosis, which compromise the biomechanical properties of the skeleton [4, 5]. These type of fractures result in a significant reduction in the quality of life and a major economic burden for our health care system as indicated by a considerable number of Disability Adjusted Life Years (DALYs) [6].

The sacrum and the sacroiliac (SI) joint being the anatomical connection between the spine and the pelvis as well as the lower limbs, its stability is crucial for the patient’s ability to walk and stand [7]. Sacroiliac screws are considered a highly popular way to stabilize the vertical instable pelvis [8]. With an anatomical correct fixation of the joint, pain and functionality can be improved. The sacroiliac corridors underlay anatomical differences in each patient. S1 or S2 screws need to be placed with high precision aiming for the central point of either the S1 or S2 vertebrae, showing the highest bone density in the sacrum. The narrowest section can be found on the level of neuroforamina therefore presenting the highest risk of cortical perforation. Sacral dysmorphism seems to affect the S1 vertebrae more often than the S2, making the S2 corridor available more often. However, the S2 vertebrae being flatter and narrower, S2 screw placement is more challenging and requires high expertise and experience. Additionally, visualization can be more challenging if a full orthogonal view of S2 cannot be achieved [7]. In this case, new methods of visualization of the pelvic bone and the drilling channel could be particularly useful.

As the patient population ages, there is a growing demand for minimally invasive techniques in pelvic surgery [9, 10]. Surgical approach-related complications in pelvic surgery have been reported at rates of up to 31% [11]. Navigation systems are essential to minimize surgical exposure and guide the surgeon, especially during limited visibility into the operative area. Computed tomography (CT)-based navigation can provide valuable information for the surgeon. Typically, optical markers are attached to the patient’s bones, and a CT scan is used for recognition and matching. The position of each instrument relative to the pelvis is then displayed on intraoperative screens using identical markers on the surgical tools. In the classical technique, screw placement is done under repeated fluoroscopy [10], but even with three-dimensional (3D) navigation, the patient is still exposed to radiation. While navigated screw placement can reduce the radiation dose, it still requires detailed intraoperative 3D CT scans [12].

Augmented Reality (AR) has the ability to virtually create a user interface, which provides valuable information and thereby facilitates spatial orientation. Artificial objects and information are displayed on three primary types of devices: head-mounted displays (HMD), hand-held displays, and fixed spatial 3D monitors [13]. In surgery, HMDs are the most practical. For example, AR can project 3D models of the patient’s CT scans onto the operating table. We hypothesize that an AR-based navigation system could guide the surgeon along the correct drilling corridor for screw placement.

Abe et al. demonstrated that a percutaneous needle incision guided by AR is feasible with minimal loss of accuracy, by using a HMD in both a preclinical and clinical setting [14]. Moreover, AR can simplify the insertion of pedicle screws, particularly for less experienced surgeons. In a study involving novice surgeons, 20 out of 40 drilling trajectories guided by AR were considered highly precise, compared to only 9 out of 40 using the freehand technique. Notably, there was no significant difference for experienced surgeons [15].

The key to achieving good results using AR lies in the precise placement of the artificial models, a process known as matching. There are various methods for recognizing or assigning the pelvis to the patient's visible body surface. In a preclinical trial with plastic models, Dennler et al. used hand or voice control for placement [15]. For percutaneous matching, different matching processes are required. Sugimoto et al. used recognition of the patient’s umbilicus and nipples for matching, enabling image overlay of preoperative CT scans onto the patient with minimal loss of accuracy. A projection of the abdominal vessels and organs onto the patient was thereby implemented [16]. By using detachable markers on the human body, Elmi-Terander et al. demonstrated that AR-guided pedicle screw placement is more accurate than freehand drilling. However, this method required a recognition scan in the operating room [17].

Therefore, we hypothesize that an AR-based navigation system could guide the surgeon along the correct drilling corridor for screw placement. For this purpose, we developed a navigational system for pelvic surgeons to assist in the safe placement of percutaneous screws into the sacroiliac (SI) joint, without radiation exposure for either the surgeon or the patient. Additionally, the system was designed to eliminate the need for a recognition CT scan in the operating room, and to potentially reduce operation time.

## Material

**Fig. 1:**
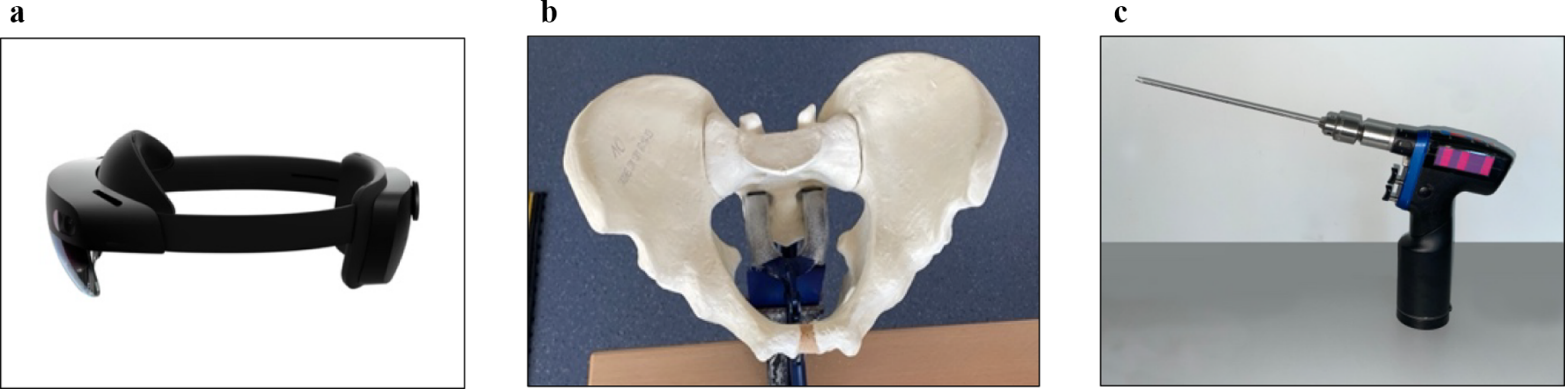
**a** Microsoft HoloLens® 2. (Image retrieved from microsoft.com). **b** Pelvic model by Sawbones® fixed in the vice. **c** Surgical drilling machine. Optical marks were attached to improve tracking by the HoloLens

Only commercially available technology was used. The Microsoft® HoloLens 2 served as the AR headset. This device (Fig. 1a) operates independently and is equipped with a see-through display [18]. The open-source game engine Unity® (Version 2021.3.3f1) was used for programming [19]. An Alienware® m15 R4 portable workstation was used for programming and implementation, supporting AR usage and development. Programming was done in C#. The tool was transferred to the HoloLens 2 via Microsoft® Visual Studio 2022 [20].

Three-dimensional pelvic models were acquired using 3DSlicer by Slicer Corp. [21, 22]. The CT scans were generated from a Sawbones® pelvic model. Ten identical models were used, with only one model being scanned by computed tomography (Fig. 1b) [23].

A SYNTHES® Colibri was used as the surgical drill. Self-designed optical markers were attached to improve object tracking (Fig. 1c). A 3D model of the drill was imported into Unity®. The model was captured using the "Polycam" application on an Apple iPhone® 14 Pro. The iPhone® 14 Pro features a LiDAR scanner for three-dimensional scanning of objects. The application is equipped with the Vuforia Engine by PTC Inc. (Version 10.15), which offers Model Target recognition. The tool utilized the depth sensor and camera of the HoloLens 2 to recognize 3D objects. The Vuforia Engine was integrated into Unity® [24].

## Methods

### Preoperative planning

**Fig. 2:**
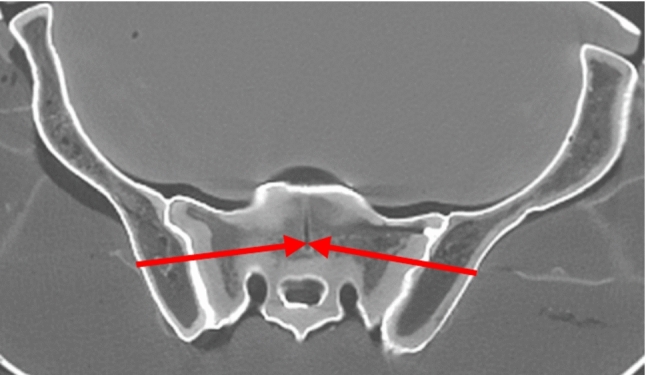
Axial CT image of the pelvis. Example for the S1 drilling corridor

**Fig. 3:**
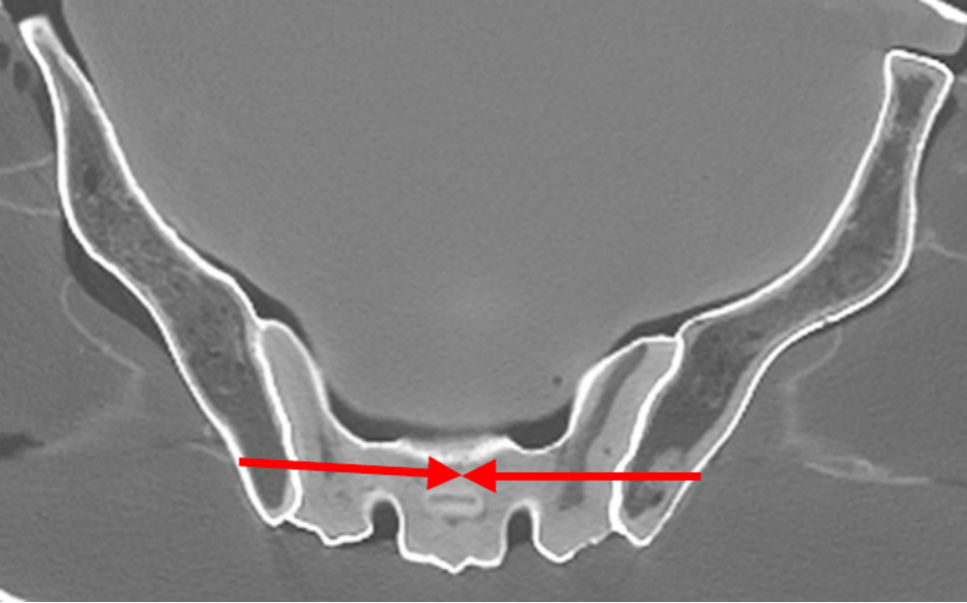
Axial CT image of the pelvis. Example for the S2 drilling corridor

Segmentation was performed using 3DSlicer, primarily utilizing the “Threshold” tool with manual adjustments as needed. The drilling corridors were planned in Slicer3D using standard 2D CT images (Figs. 2 and 3). The planned screws were integrated into the 3D segmentation of the pelvis. The segmentation, including the screws, was then exported as a single “Wavefront object” (.obj) file.

A new 3D Unity® project was created. Microsoft’s® Mixed Reality Toolkit 2 (MRTK2, Version 2.8.2) was imported into the Unity® project. This toolkit offers various templates for HoloLens 2 programming.

The 3D pelvic model was imported into Unity®. The entry points and central points of the planned drilling trajectories were marked using sphere objects in Unity®. To enable Unity® to interact with the drilling corridors, the planned corridors were converted into Unity® cylinder models. The entry points and centers of the drilling trajectories were also marked. Two S1 and two S2 trajectories were integrated.

The program was transferred to the HoloLens 2 via Microsoft® Visual Studio 2022 [20]. The transfer process took approximately 6 min and could be performed preoperatively. Including segmentation and planning, the entire process took between 45 and 60 min during the test.

### Trial procedure

The experimental series was conducted at the local trauma training center. The matching and drilling were performed by the author, who has no prior experience in pelvic surgery. The pelvic models were secured in a vice.

The matching process involved probing anatomical landmarks on the patient. Both the anterior superior iliac spines and the symphysis were marked using virtual arrows. Four screws were inserted into each pelvis—two on each side. The drilling order was as follows: S1 right, S1 left, S2 right, and S2 left. No changes were made to the matching after starting the first screw of each pelvis. Initially, a K-wire was inserted, followed by manual insertion of cannulated pelvic SI screws (Axomed, 7.5 mm cannulated SI screw).

A total of 20 S1 screws and 19 S2 screws were placed. The drilling was performed without visual access to the pelvis to minimize visual influence on the drilling direction. A paper box was used to cover the pelvic model, which was placed immediately after marking the anatomical landmarks. As a result, the final matched position could not be visually checked for inaccuracies. An integrated stopwatch measured the time required for matching and drilling each screw.

After the insertion of four screws, the positioning was verified using computed tomography (CT). After imaging, the screws were removed to be reused for the next pelvis.

In a subsequent test, the developed navigational system was tested on human cadavers. The test was conducted on a single cadaver acquired from the local institute for clinical anatomy. Two S1 and two S2 screws were placed. The drilling was performed by the same operator as in the previous tests. Screw placement was verified using CT.

For all drilling procedures, the direction of cortical perforation was evaluated for each drilled screw. A correct placement was defined by the absence of perforation. A perforation was generally defined as a breach of the cortical layer of the pelvic bone. An anterior perforation was defined as a screw reaching into the small pelvis. A superior or inferior perforation was defined as a breach into the adjacent neuroforamina. A posterior perforation was defined as a breach into the spinal canal.

Besides perforation, the maximal lateral deviation (MLD) was calculated. The MLD is defined as biggest orthogonal distance of any point on the drilled axis from the planned axis. Additionally, deviation of entry and central aim point, as well as angular deviation was measured.

### Intraoperative workflow

**Fig. 4:**
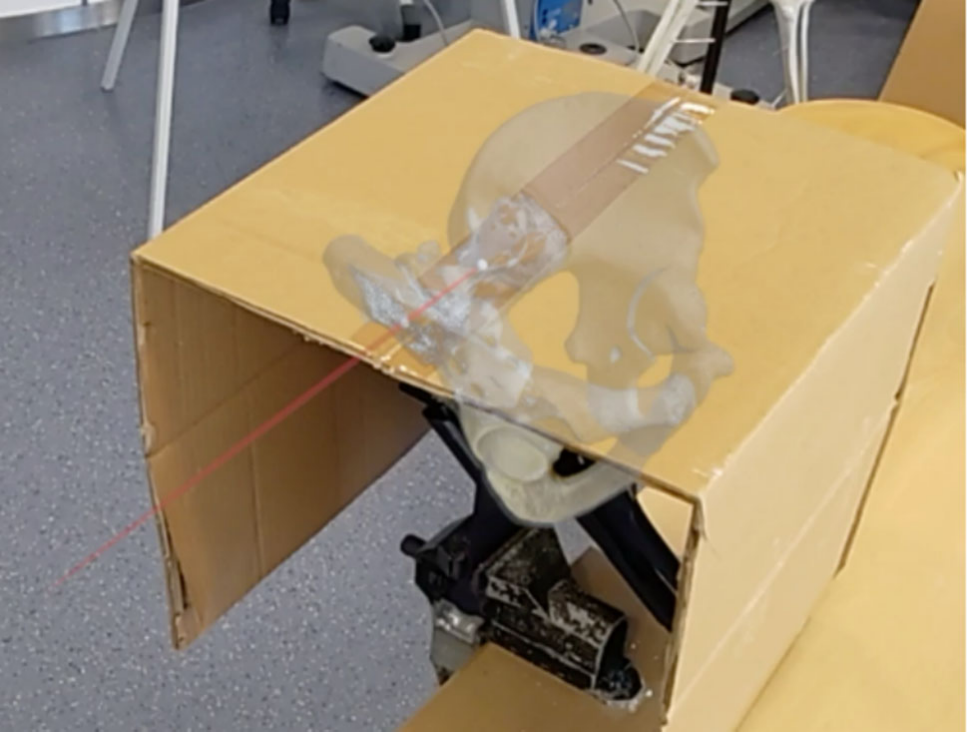
In-game picture of matched Pelvis. Visibility set to 50%

**Fig. 5:**
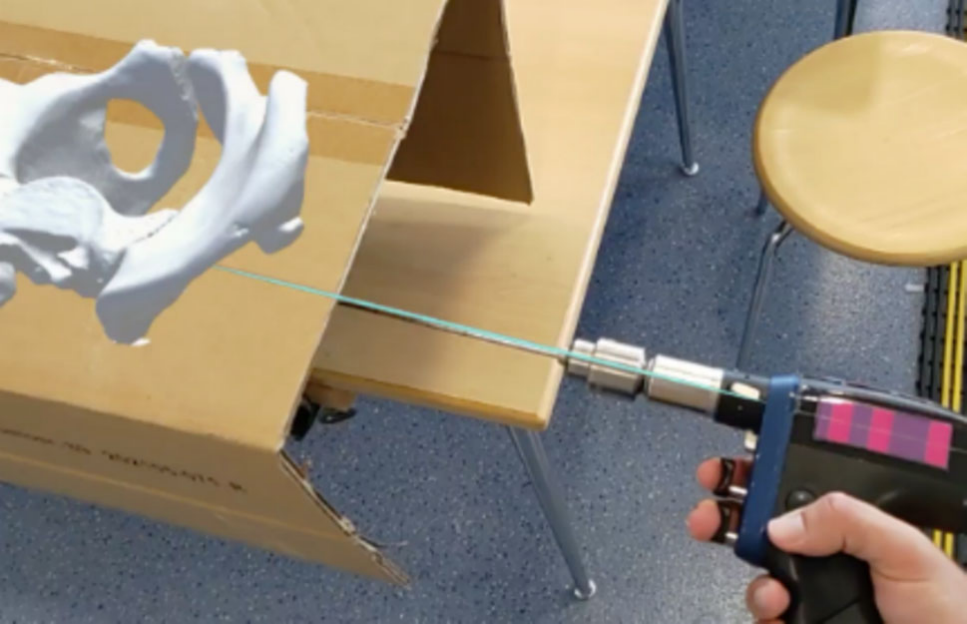
In-game picture of artificial laser in blue. Visibility of the pelvis set to 100%

The user was guided through the matching process of the pelvis. Once all three points were marked, the pelvis was automatically resized and aligned. The system tried different positions until the most accurate alignment was found. The pelvis was continuously displayed unless otherwise specified (Fig. 4). The transparency of the pelvis could be adjusted to 50% or made invisible. This adjustment only affected the pelvis itself (Fig. 5). The drilling trajectory was always visible unless turned off in the menu. This feature allowed the surgeon full visibility of the surgical field.

Navigation could be activated using either the menu or voice control. The drilling corridor was displayed to the surgeon. After completing the insertion of one planned screw, the next pre-planned screw corridor would appear.

Tracking of the surgical drill could also be activated via the menu or voice control. Once 3D model recognition of the surgical drill was successful, an artificial laser appeared overlaying the drill head (Fig. 5). The surgical drill could then be positioned to match the planned drilling corridor. Continuous optical focus of the drill was required for ongoing tracking. Moving the drill in and out of the field of view helped in case of any visible discrepancy between the artificial laser and the real drill head. The entry point turned green if the drill was positioned correctly. If the central point of the drilling trajectory was reached, the drilling axis turned green as well. The artificial laser could be toggled on or off using the menu.

The navigation could be reset using either voice control or the menu.

### Statistical analysis

Data were collected and analyzed using IBM® SPSS software (Version 20.0.2.0). An exploratory data analysis was conducted to visualize the MLD, deviation from the entry point and central aim point, as well as the angular deviation. The time required for each screw placement and matching was also examined. Significant differences between S1 and S2 screw placements were evaluated. A Shapiro–Wilk test was performed to check for Gaussian distribution in all measured variables. A Mann–Whitney U test was used for maximum lateral deviation and for entry and central aim point deviation due to the non-Gaussian distribution. A t-test for unpaired data was applied for the angular deviation, assuming Gaussian distribution. The level of significance was set to *p* < 0.05. All 10 pelvises were included in the analysis. In one case, the system crashed after the placement of 3 out of 4 screws, and the last screw could not be placed; therefore, it was not included in the analysis. Time measurement could not be performed properly for all four screws in that case. No outliers were removed from the data.

Similar analyses were conducted for screw placements in the human cadaver.

## Results

A functional AR tool was successfully developed. Proper matching was achieved with our system. The median time for the matching process in our test series was 3 min and 6 s. The median duration for S1 screws was 6 min and 40 s (5 min 19 s–7 min 26 s) while the median duration for S2 screws was 4 min and 26 s (4 min 16 s–5 min 32 s). The procedural duration included both machine drilling and manual screw placement. A decrease in procedure duration was observed throughout the test series.

No significant differences were found between the maximal lateral deviations and the deviations in angle and entry points for S1 and S2 screws. However, a significant difference was found between the deviations of the central aim points for S1 and S2 screws. Thus, deviations were analyzed separately for S1 and S2 screws.

**Fig. 6:**
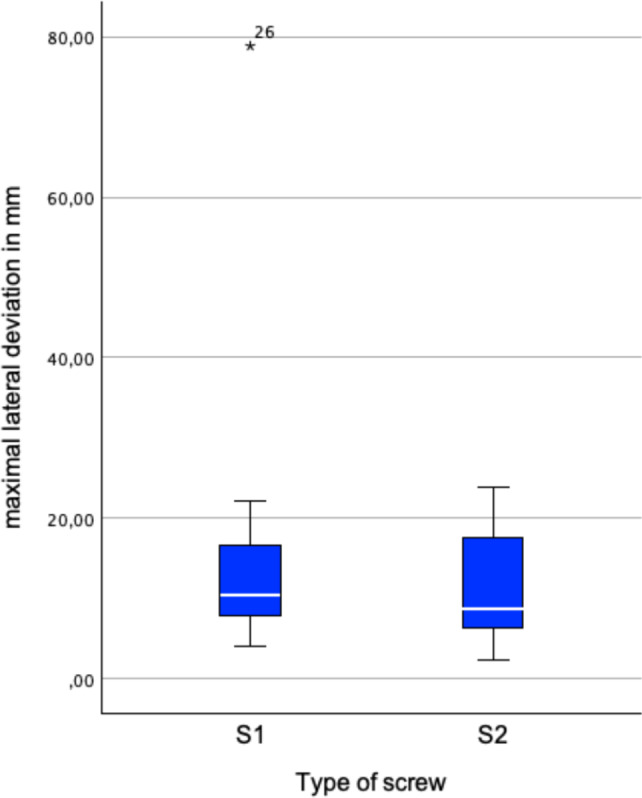
Maximum lateral deviation (MLD) from the planned drilling axis. There was no significant difference for S1 and S2 screws

**Fig. 7:**
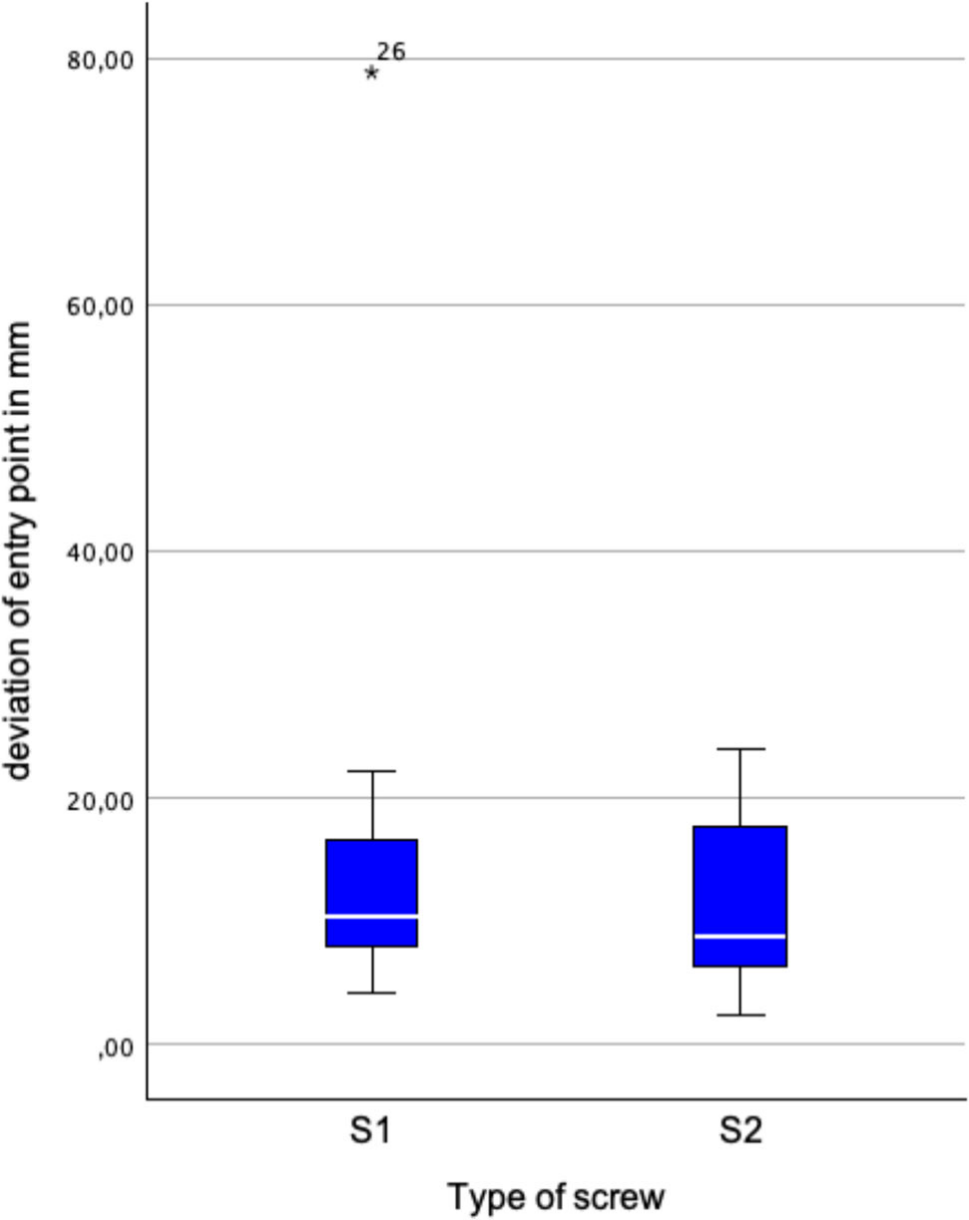
Deviation from the planned entry point. There was no significant difference for S1 and S2 screws

**Fig. 8:**
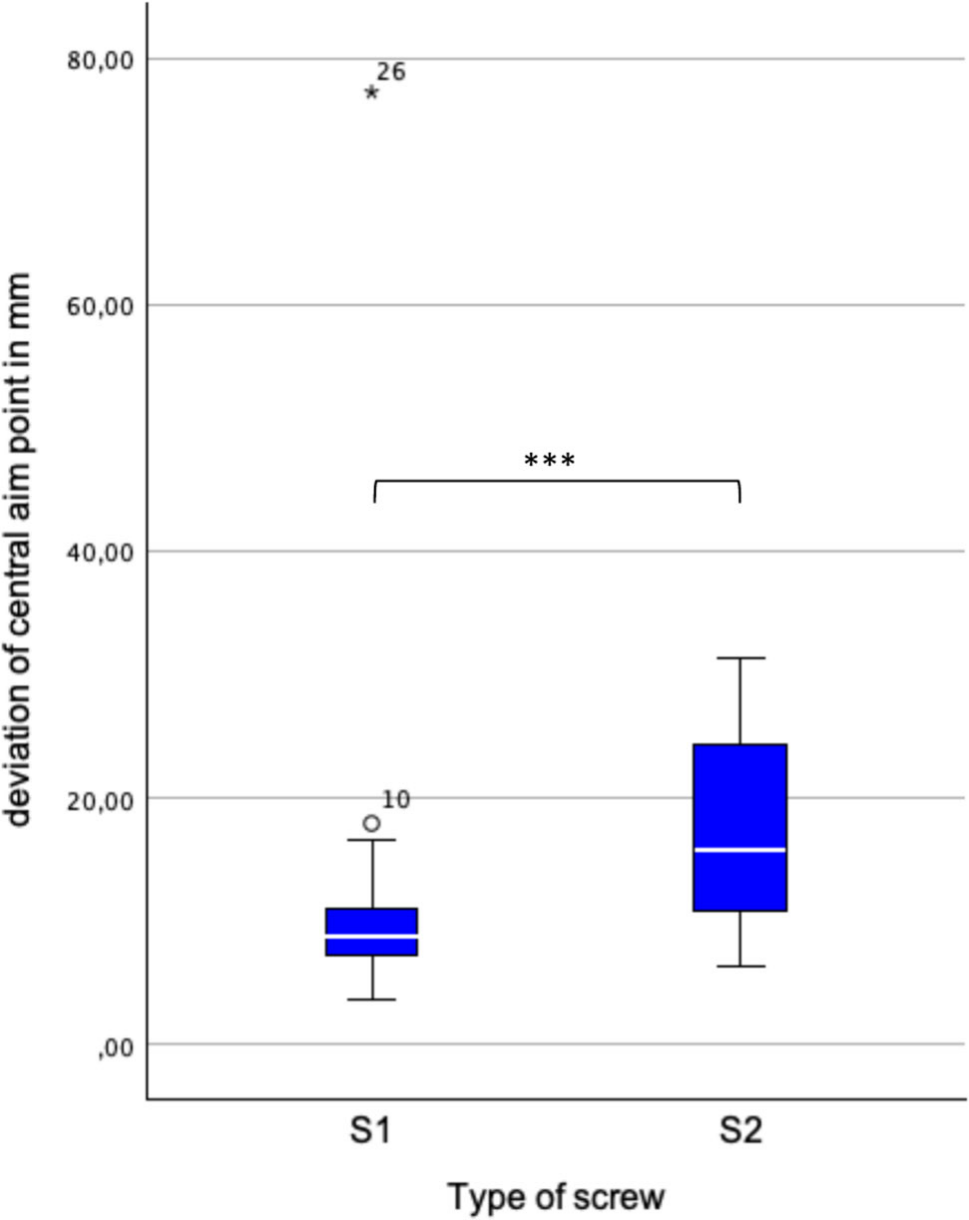
Deviation from the planned central aim point. The central aim point was hit more precisely for S1 screws

Eighteen out of 20 (90%) S1 screws and 3 out of 20 (15%) S2 screws were placed correctly. S1 screws were placed significantly more accurately (*p* < 0.001). The median of MLD was 10.0 mm (7.7–14.1 mm) for S1 and 10.6 mm (5.6–16.8 mm) for S2 screws (Fig. 6). The median deviation from the entry points was 10.4 mm (7.9–16.6 mm) for S1 and 8.7 mm (6.4–17.6 mm) for S2 (Fig. 7). The median deviation from the central aim points was 8.7 mm (7.1–11.0 mm) for S1 and 15.8 mm (10.8–24.4 mm) for S2 (Fig. 8). The central aim point was reached significantly more precisely with S1 screws (*p* < 0,001).

**Fig. 9:**
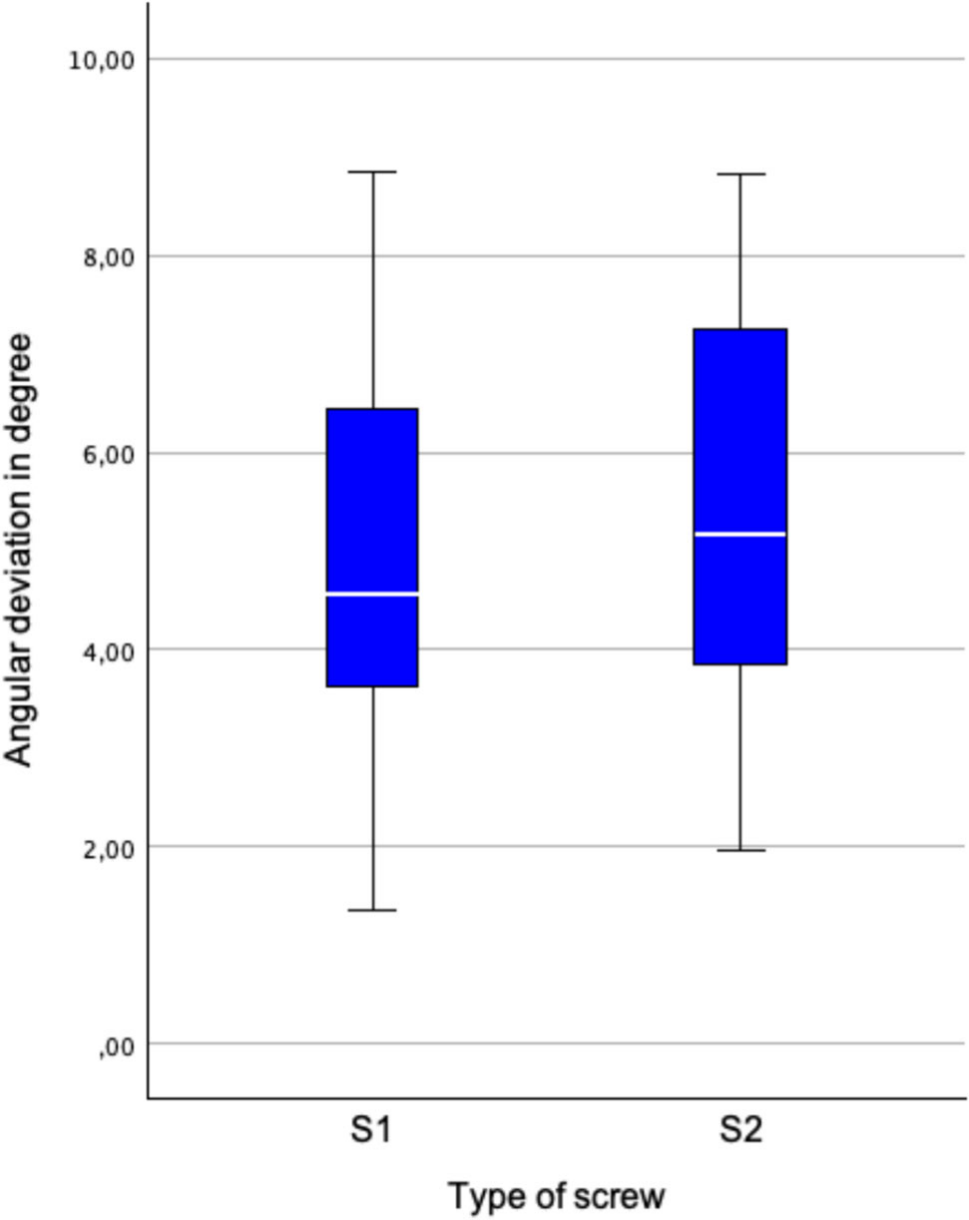
Angular deviation of screw placement. There was less angular deviation for S1 screws

The median angular deviation of the screws was 4.6° (3.6°–6.5°) for S1 and 5.2° (3.8°–7.3°) for S2 (Fig. 9).

### Cortical perforation analysis

**Fig. 10:**
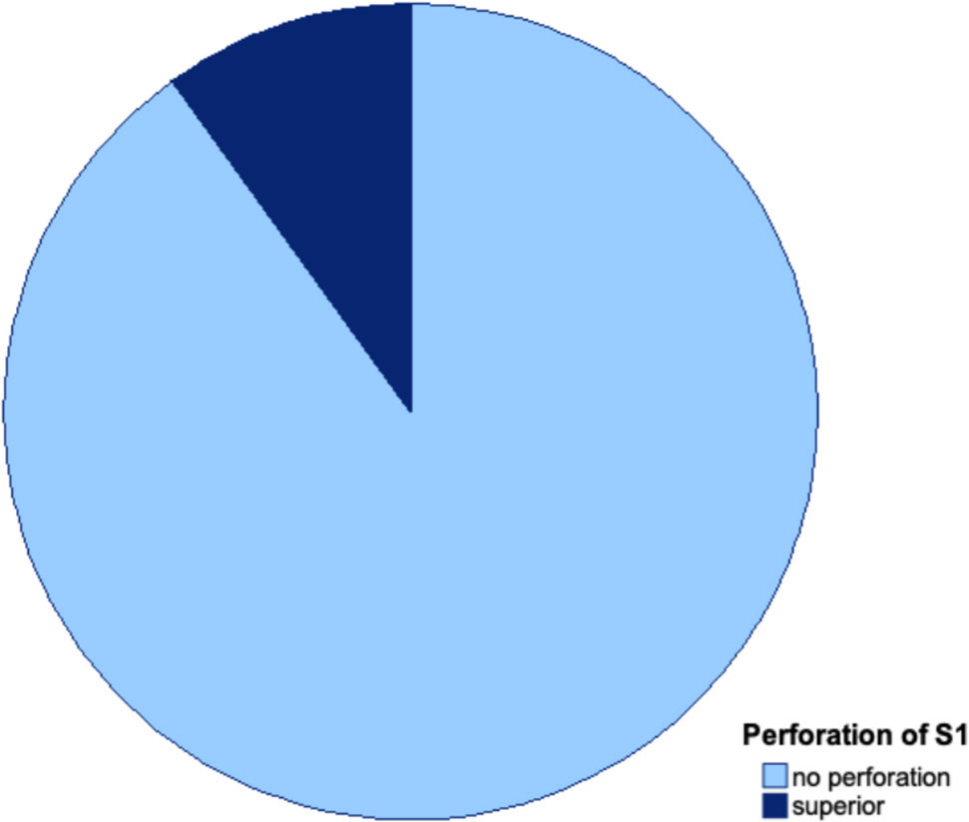
Direction of periosteal perforation for S1 screws. Mostly there was no perforation

**Fig. 11:**
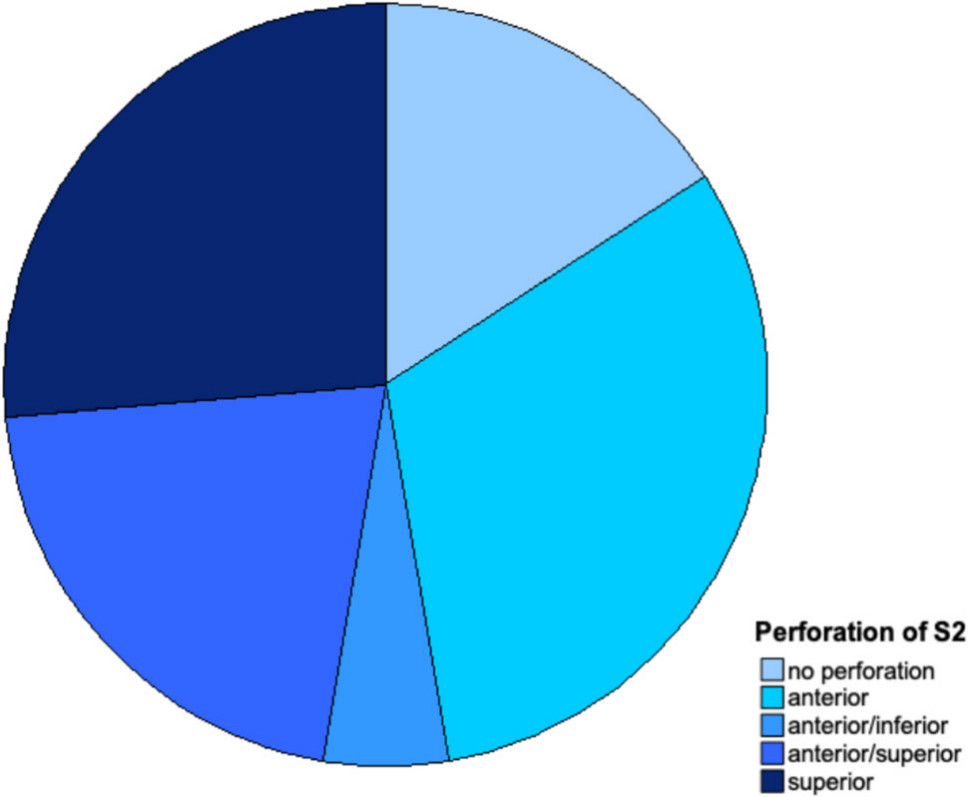
Direction of periosteal perforation for S2 screws. A broader spectrum of directions can be noticed compared to S1 screws. Isolated or combined perforations occurred. There was no posterior perforation

The anatomical direction of cortical perforation was analyzed. In most cases, there was no perforation. Among perforated cases, most involved a single anterior or superior perforation. Some combined anterior/inferior and anterior/superior perforations were observed. No posterior perforations occurred. For S1 screws, only two cases of superior perforation were observed (Fig. 10). For S2 screws, multiple perforations were observed, with the most common being anterior perforations into the small pelvis (Fig. 11).

### Human cadaver test

**Fig. 12:**
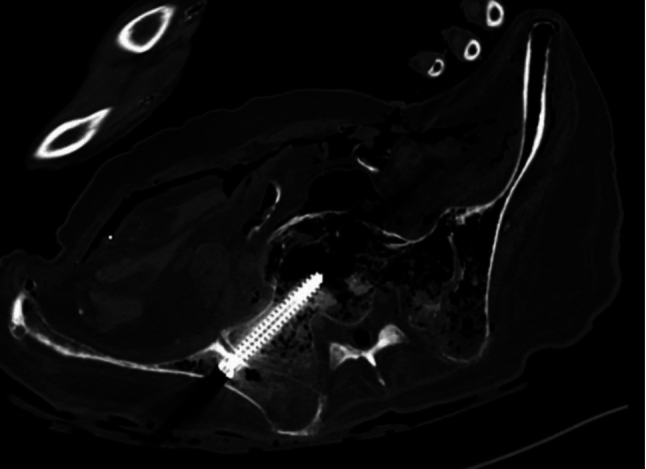
CT scan of correctly drilled S1 screw in human cadaver using the AR-guided navigation

The consecutive test in a human cadaver was successfully completed (Fig. 12). Both S1 screws were placed correctly, but no S2 screws were placed correctly. The mean MLD for S1 was 10.6 mm and 17.2 mm for S2. Maximal MLD occurred in both S1 screws at the base, whereas for both S2 screws at the tip. The median deviation from the entry points was 10.1 mm for S1 and 15.8 mm for S2. The median deviation from the central aim point was 8.9 mm for S1 and 18.5 mm for S2. The median angular deviation was 3.5° for S1 and 4.0° for S2. The cortical perforations of the S2 screws were observed in the anterior and superior-anterior directions.

## Discussion

During the development of our own navigation system, we encountered several challenges that we had to overcome. The system demonstrated sufficient accuracy for the S1-screw corridor, which was wider compared to the S2-screw corridor in the pelvic models used for this study. However, reliable placement of S2 screws in the narrower corridor was not consistently achieved.

### Limitations

Designing the study, no control group was implemented. The experiment was done as feasibility study with the aim to show possible AR-guidance for SI-screws. The lack of a control group must be seen as limitation of the study.

Several aspects of the system need further optimization. The primary issue was the optical stability of the holograms. Even though we used Microsoft® HoloLens 2's integrated stabilization system, the pelvis still exhibited optical instabilities, a phenomenon known as "swimming." This issue primarily occurred when the user moved around the pelvis [25]. When positioning the drilling machine, a continuous movement around the pelvis was required to ensure a correct drilling direction from every angle. The "swimming" of the pelvis made it difficult to align the drilling machine precisely with the planned drilling trajectory. There are different features which could be implemented in future versions such as Spatial Anchors and the Microsoft® World Locking Tool which can decrease “swimming”. Spatial Anchors can increase hologram stability by locking the hologram’s position to the surrounding. Additionally, the matching arrows could be locked to the patient’s skin, rather than guessing the depth of the bony matching points.

The deviations from the planned corridors could be attributed to two primary factors. The first was the aforementioned "swimming" of the artificial pelvis. The second was likely the imprecision of the matching process itself. Marking the anatomical landmarks was not always possible with high precision and depended on the user’s accuracy.

It is challenging to pinpoint whether one or both of these factors contributed to the inaccuracies observed. However, when reviewing the results for each pelvis separately, we made some observations. Seven out of the ten pelvises had more than two perforations. In these pelvises, the direction of perforation of the screws was of particular importance. In five of these seven cases, all screws had approximately the same direction of perforation, which suggested that the matching process may have been inaccurate. The remaining five pelvises either had only one perforation or showed differing perforation directions, pointing towards potential issues with the movement of the pelvis or user error. Notably, the drilling was performed by a medical student without specialized training in trauma or pelvic surgery, which might have influenced the results. A more accurate determination of the cause of the inaccuracies was not possible in this trial.

The idea was matching the pelvis without additional trauma for the patient. Still, only three matching points could be not sufficient making the matching too susceptible to errors. Adding additional matching points should be evaluated as improvement method for matching. The umbilicus or the major trochanters could be used. Besides expanding the matching method, an error feedback loop could be added to future versions, for example checking of accuracy by using surface recognition of the patient’s skin. Therefor segmentation must be done with the skin surface of the patient as well as the pelvic bone. In case of insufficient matching, it could be redone eliminating systemic errors.

It remains essential to determine why there was a significant difference in the accuracy of positioning the screws with a less correctly placed S2 screw. The general sources of inaccuracy, such as "swimming" and potential matching deviations, affect both screw types.

The system establishes a virtual plane by aligning the pelvis based on three anatomical landmarks. Generally, the greater the distance between these landmarks, the lower the risk of angular misalignment. Compared to the distance between the iliac spines, the symphysis is positioned closer to the axis formed by the superior iliac spines, making the plane more susceptible to tilting around this axis. In 11 out of 16 cases (69%), S2 screws exhibited perforation with an anterior component, suggesting a rotational error in the matching process. If the symphysis was marked too far anteriorly, this could account for the observed perforation. S1 screws, being located closer to the described axis, were less affected by rotational errors. Furthermore, the available space for the S1 screw tip before perforation occurs is larger than that for S2 screws, reducing the likelihood of perforation. This phenomenon could also be observed in the cadaver experiment, showing two anteriorly perforated S2 screws.

Interestingly, there was no significant difference in MLD found for S1 and S2 screws. Cortical perforation of sacroiliac screws mostly occurs in the middle section as the canal usually is the tightest there. In our series, the MLD either occurred at the base or the tip of the screw. In S1 screws, 13 out of 20 (65%) occurred at the base of the screw. For S2 screws, 17 out of 19 (89%) occurred at the tip of the screw. Regardless the location of the MLD, MLD was not significantly different for S1 and S2 screws (*p* = 0.74). 14 out of 21 (66%) correct screws had the MLD at the base, whereas 17 out of 18 (94%) of misplaced screws had the MLD at the tip. This leads to the assumption that the location of MLD determines whether the screw is placed correctly or not, not the MLD itself which doesn’t show a significant difference between the screw types. If the MLD is located at the base, it is mostly influenced by the entry point whereas a severe MLD of the tip consists of deviation of entry point and angular deviation. S2 screws also showed significant higher deviation of the central aim point. This deviation also takes the drilling depth into account. A low central MLD and low central aim deviation seems to be crucial for correct placement. Correct placed screws, regardless of screw type, showed significant lower MLDs (*p* = 0.027).

In one case, a system crash forced the termination of screw placement. The cause of this failure remains unclear. While the system can be restarted without compromising sterility on the operating table, the matching process must be reestablished. Given that the patient is draped for sterility, this can be challenging in a clinical setting. Therefore, it is crucial for the surgeon to be prepared to switch to a traditional navigation technique, even though such an event occurred only once during the experiments.

### Advantages

Despite these challenges, we were able to demonstrate that even if the drilling corridor was not perfectly aligned, proper drilling of S1 screws was still achievable. This was primarily due to the ability to maintain visibility of the pelvis throughout the procedure. Considering the user’s level of expertise, the results for S1 screws were deemed satisfactory. Misplaced sacroiliac screws are reported to occur in up to 23% of cases using the traditional fluoroscopy method [26]. By employing newer CT-based navigation systems, misplacement rates of 95–100% can be achieved, but these systems rely on intraoperative CT scans for navigation matching, a time-consuming and complex process requiring significant team expertise.

In a preclinical trial, Heining et al. also explored AR-guided insertion of pelvic and acetabular screws. Using surface recognition, the virtual pelvis was aligned with the pelvis model. They drilled 12 corridors into each of 8 hemipelvises. Of the 96 pathways, 83 (86.5%) were correctly completed. The mean deviation at the entry point was 3.99 ± 1.04 mm and 4.8 ± 0.8 mm at the endpoint. The angular deviation was 4.3 ± 1.8° [27]. Similar rates of correct screw placement for S1 screws could be achieved in our experiments even with lesser accuracy. Additionally, our system can be transferred into a clinical setting as shown in the cadaver experiment whereas surface recognition of the pelvic bone is not feasible in a human patient.

Our system used Vuforia, which leverages the HoloLens cameras and depth sensors to track 3D objects. However, Vuforia’s accuracy was insufficient for precise drilling guidance. The user had to manually align the drilling head with the intended trajectory. This was achieved by adjusting the transparency of the artificial drilling corridor to 50%, allowing the user to clearly see both the real drill and the virtual corridor. The model tracking primarily served to control drilling depth, and thus, it was deemed sufficient for this purpose.

A preoperative planning is possible and took around 45–60 min by an experienced user in our series. Pelvic fractures usually not being operated in an emergency setting, this could be done days or hours in advance of the operation as soon as a CT scan is acquired. In the future, automatic segmentation and semi-automatic transfer to the HoloLens should be implemented, simplifying and fastening the current workflow. Implementing automatic processes could not only shorten preparation time but also assist inexperienced users in the process.

The system operated completely radiation-free, eliminating the need for additional fluoroscopy in the operating room. The non-invasive approach spared the patient from additional trauma associated with the insertion of navigational markers. The system relied on a preoperative CT scan of the patient, reducing both the patient's and the medical staff's exposure to radiation during the procedure. The time required for the procedure was also significantly shorter compared to standard navigation tools or fluoroscopy. With a median time of approximately 10 min, including a 3-min matching process and a 5–7 min screw placement, our system could significantly shorten the operation time. In contrast, traditional pelvic screw placement can take up to 69 min per screw [28]. It should be noted that the measured time did not include cutaneous preparation and closure. Moreover, the adaptation of the user to the system was evident from the decreasing operation time throughout the trials, suggesting that the system is user-friendly and easy to learn.

### Comparison

Unlike traditional methods, our system did not require intraoperative adjustment of a C-arm before drilling. Instead, X-rays taken after the guide wire placement and at the end of the procedure could suffice for verifying the results. The system's goal is to use X-rays primarily as a tool for controlling and documenting the results, rather than for navigation during the procedure.

AR-guided pelvic screw placement has not found its way into clinical practice yet. Percutaneous screw placement in pelvic surgery is usually executed either using standard fluoroscopy or CT-based intraoperative navigation.

Zwingman et al. conducted multiple studies comparing standard fluoroscopy with CT-based navigation systems for percutaneous sacroiliac screw placement. While no significant differences in operative time were observed between the techniques, CT-based navigation significantly reduced radiation time (*p* = 0.0003) and radiation dose (*p* = 0.001). Screw placement accuracy was evaluated based on cortical perforation grading, with Grade 0 indicating optimal placement without perforation. Navigated screw placement resulted in a significantly higher proportion of Grade 0 screws (*p* = 0.02) [29]. Additionally, intraoperative and postoperative complication rates were analyzed using data from the German Pelvic Trauma Registry, revealing no significant differences between the navigation methods for intraoperative (*p* = 0.42) or postoperative complications (*p* = 0.6542) [30]. The comparison is visualized in Table 1 and set against our developed navigation system.

**Table 1:** Comparison of conventional 2D-fluoroscopy and CT-based navigation for percutaneous SI-screw placement [29–31]

	Characteristics [31]	Operation time per screw (min) [29]	Radiation time per screw (s) [29]	Correct placement (no cortical perforation) [29]	Complications rates (intra- and postoperative) [30]
Conventional 2D-fluoroscopy	High expertise and experience required Only one image at a time Difficult radiographic interpretation Variations in the anatomy of the posterior pelvis Standard fluoroscopy can be limited by intestinal gases or obesity	69 (± 38)	141 (± 69)	40%	**Intra**: 8/83 (9.6%) **Post**: 29/83 (32.5%)
CT-based navigation	Matching of preoperative scans and patient Markers for optical tracking usually required Good visibility of drilling channel	72 (± 16 min)	63 (± 15)	69%	**Intra**: 4/39 (10.3%) **Post**: 13/39 (33.4%)
Self-developed AR-based navigation system	Intuitive handling Preoperative planning process Intraoperative matching is not dependent on additional imaging Visualization of drilling channel onto the operational field	6 (± 2 min)	No intraoperative radiation needed	**S1:** 90% **S2:** 15%	

In comparison to other navigation systems, our AR tool is more cost-effective. After the initial investment in hardware, ongoing costs are minimal.

### Future work

In the subsequent test using a human cadaver, the system showed promising results. The correct placement of S1 screws was achieved, consistent with preclinical trials. The drilling procedure, performed by the same user from the preclinical test, demonstrated that pelvic screws could be successfully placed in a human body even by an inexperienced user guided by our system. It should be noted that the results may vary depending on factors such as the thickness of the patient’s soft tissue. In the cadaver used in this trial, probing of the anatomical landmarks was straightforward. As the drilling was done only in one cadaver, the results must be interpreted in this limited setting. To evaluate the influence of different patients’ soft tissue and prove the feasibility of AR-guided SI-screw placement with our system, consecutive cadaver studies with a larger cohort would be advisable.

## Conclusion

In this in vitro study, our tool enabled accurate and reliable visualization and alignment of the human pelvis using Augmented Reality. The radiation-free AR navigation system demonstrated high precision for S1 sacroiliac screw placement, even among inexperienced users. Lower accuracy for S2 screw placement and some hologram instabilities were observed, highlighting specific areas for optimization. Overall, the promising results underscore the system’s strong potential and lay the groundwork for further technological refinement and future clinical application.
